# Pathways to change in existential group treatment: experiences from older adults with psychological distress in primary care

**DOI:** 10.1186/s12877-025-06157-4

**Published:** 2025-07-08

**Authors:** Isak Erling, Carl Anton Waltersson, Margda Waern, Maria Tillfors, Sara Hed, Stefan Wiktorsson, Anne Ingeborg Berg

**Affiliations:** 1 Department of Psychology, University of Gothenburg, Gothenburg, Sweden; 2 Department of Psychiatry, Institute of Neuroscience and Physiology, University of Gothenburg, Gothenburg, Sweden; 3 Department of Social and Psychological Studies, Karlstad University, Karlstad, Sweden; 4 Department of Psychotic Disorders, Sahlgrenska University Hospital, Region Västra Götaland, Gothenburg, Sweden; 5 Department of Neuropsychiatry, Sahlgrenska University Hospital, Region Västra Götaland, Gothenburg, Sweden; 6 Slottsskogen Primary Care Center, Närhälsan, Region Västra Götaland, Gothenburg, Sweden

**Keywords:** Existential therapy, Existential group treatment, Pathways to change, Older adults, Psychological distress, Experiential avoidance, Qualitative analysis

## Abstract

**Background:**

There is a need for age-adapted treatments for older adults with psychological distress and existential therapy could be of particular relevance for mental health issues related to aging. The aim of this study was to understand the process of existential therapy for older adults by exploring how pathways to change are expressed in experiences from existential group treatment.

**Methods:**

Seventeen participants aged 75 and above with psychological distress took part in individual interviews after completion of a 7-week existential group treatment in primary care. The participants were recruited from five primary care centers in the Västra Götaland Region of Sweden. Data were analyzed using thematic analysis in relation to pathways to change in Wampold’s Contextual Model of Psychotherapy.

**Results:**

The analysis resulted in six themes. Central themes in understanding pathways to change in existential group treatment for older adults were Dare to talk about life and death, Letting myself be old, and Making the most of life despite difficulties. When recognizing things as they are and being able to face the reality of being old without avoidance, a wish to make the most of life despite difficulties emerged. Perspectives on the therapeutic process also included Commitment matters, Engaging with others and Developing new and different relationships. These themes captured the participants’ experiences of choosing to commit to treatment and the relational process of engaging in existential group treatment.

**Conclusions:**

Older adults’ experiences of existential group treatment suggest that engaging in existential dimensions of aging can be helpful when done in a structured setting together with others. Reduced experiential avoidance, increased determination to do what feels meaningful, relational aspects as well as one’s own commitment to therapy can help explain how existential group treatment may contribute to change among older adults with psychological distress.

**Supplementary Information:**

The online version contains supplementary material available at 10.1186/s12877-025-06157-4.

## Background

Symptoms of depression and anxiety are common in older adults [1] and high suicide rates are observed in this age group in many countries around the globe [2]. Despite the well-documented need for relevant mental health treatments, older adults are often underrepresented in utilization of mental health services [3, 4]. Ageist attitudes, both societal and internalized, have been widely documented, with the risk of contributing to inadequate care for older adults and negative health consequences [5, 6]. In Sweden, access to psychological treatments for older adults remains low and these unmet needs were recently highlighted by the Swedish Board of Health and Welfare, with a call for new treatments adapted for this age group [7].

Existential problems of life such as loneliness and coping with the approach of death and parting have been proposed as central challenges in old age [8]. Interest in these issues is growing and was further catalyzed by the covid-19-pandemic [9, 10]. Existential concerns such as meaning, loneliness and death anxiety have been linked to subjective wellbeing and psychopathology [11–13], contributing to a growing interest in existential therapy as a potential treatment for older adults with psychological distress. Several studies [14–16] have documented a need and desire from older adults to get the opportunity to engage in existential dialogs with their professional caregivers about their mental health conditions. In a focus group study with older adults who had pharmacological treatment for depression [14] a clear message was that the participants wanted to talk about the existential dimensions of aging and death. Participants saw death not only as a threat, but also as a potential source of escape from mental pain. The latter echoes results from studies on older adults’ reasons for attempting suicide [15, 16], highlighting the need for older patients with mental health issues to have opportunities for a deeper dialogue with professional caregivers about the existential concerns they experience in daily life as they age. The ability to address existential concerns has been proposed as central to identity development in old age [17] and there has been a call to incorporate the existential perspective into health services for older adults, as well as in the development of new interventions for this age group [18]. With primary care being the frontline of the medical care system, the integration of existential interventions tailored to older adults within this setting may be particularly relevant in delivering meaningful public health benefits.

While existential therapy could be of particular relevance for mental health problems related to aging, we are aware of no published study in which existential therapy has been evaluated exclusively for older adults with psychological distress in primary care. Existential therapy has been described as ‘a rich tapestry of intersecting practices’, generally understood as a sub-approach within the broader category of humanistic therapies [19]. Over time, it has come to be defined as a distinct form of therapy that has the human condition itself as its central focus, and aims to illuminate how each unique individual choose their way of being in the world, ultimately confronting some of the most fundamental questions regarding human experiences [20]. Qualitative methods have been considered an important type of research in understanding the complexity of human experiences in existential therapy with a special potential of bringing in-depth insights into individuals’ lived experiences, therapeutic processes, and ways of finding meaning through therapy [21]. Qualitative studies have explored the role of existential concerns in relation to illness [22] and ways of coping with illness [23, 24], as well as patients’ experiences of particular existential therapies [25]. Research on existential group interventions has documented existential sharing, togetherness and acceptance as vital components of the therapeutic process [26, 27]. Additionally, helping clients to accept and increase their sense of meaning in life despite life’s inevitable challenges has been described as key mechanisms of change in existential therapy [28]. Although there are some indications for processes in existential therapy that might be helpful for older adults with psychological distress, there is still limited knowledge about older adults’ experiences of existential therapy and their perspectives on the therapeutic process.

### Pathways to change in psychotherapy

Building on the question *if* therapy works, much of contemporary psychotherapy research is nowadays also exploring the question *how* therapy works [29, 30]. In this context, several models have been developed to understand how change occurs in therapy, emphasizing both the client’s readiness to change through distinct stages within a behavior change process, outlined in the transtheoretical model [31], as well as the influence of therapist and client characteristics on treatment outcome, captured in the four-factor model of change [32]. Expanding on these themes, Wampold and colleagues have investigated how psychotherapeutic outcomes can be understood in relation to factors such as therapeutic relationship, therapist- and client factors as well as specific techniques from different psychotherapeutic practices [33]. In understanding how psychotherapeutic change may occur, Wampold has developed the Contextual Model [34], which stresses the potential of psychotherapy to produce symptom reduction and better quality of life among patients via three different pathways to change. Although each pathway to change, to some extent, may be therapeutic in itself, Wampold stresses the need to understand psychotherapeutic change in a context where all three pathways to change are affecting the process of how a particular form of therapy works. The three pathways to change in Wampold’s Contextual Model are (1) Expectations (2) A real relationship and (3) Specific ingredients in therapy, and can be understood as a framework for understanding how change may occur in a specific form of therapy [34].

With a need for new treatments adapted for older adults that emphasize the relation between existential dimensions of aging and mental health, this study aspires to fill a gap in the understanding of how existential therapy may work for older adults with psychological distress.

### Aim

In relation to Wampold’s Contextual Model stating that therapeutic change can be understood from three pathways in the therapeutic process, this study aims to understand older adults’ experiences of existential therapy with the research question: How are pathways to change expressed in older adults’ experiences of existential group treatment?

## Method

### Participants

This study included 17 participants (14 women and 3 men) with a median age of 79 years (range 75–84) from five primary care centers in the Västra Götaland Region, Sweden. All participants were recruited among primary care patients in an ongoing research project, “Existential group treatment for older adults (75+) with psychological distress in primary care: a randomized controlled trial” [35]. To be included in the trial, all participants had self-reported psychological distress, as defined by a score of ≥ 3 on the twelve-item General Health Questionnaire (GHQ-12) [36]. This score indicated that their general health was worse than usual or much worse than usual in at least three areas prior to treatment, such as for example feeling unhappy or depressed, feeling unable to overcome difficulties, and loss of confidence in oneself. Exclusion criteria were ongoing severe alcohol use disorder, ongoing post- traumatic stress disorder, ongoing psychotic or manic episodes, acute suicide risk, or other mental health problems necessitating the offering of other specified treatment as identified by the Mini-International Neuropsychiatric Interview (M.I.N.I.) [37]. Exclusion criteria were also clinical diagnosis of dementia or a score of ≤ 25 on Mini-Mental State Examination (MMSE) [38]. Among those who took part in the current qualitative study, all had completed the seven-session existential group treatment at their respective primary care center in 2023 during the first round of the randomized controlled trial. 14 participants attended all seven sessions, two participants missed one session, and one participant missed two sessions.

Fifteen participants were born in Sweden and two were from non-Nordic countries. Most participants (*n* = 13) lived alone. Eight of the participants were widowed, six were divorced and three were married. Eleven participants reported having ≥ 14 years of education, five had 10–13 years, and one had ≤ 9 years. Occupations prior to retirement were mixed but reflected the relatively high level of education. All but two participants reported ongoing physical problems that seriously affected everyday life in accordance with the Cumulative Illness Rating Scale-Geriatrics [39]. Nine had problems with joints, muscles, and skeleton, four had conditions of the lower gastrointestinal tract, and four had problems related to eyes, ears, nose and throat. Four participants reported a history of cancer. Eight participants reported taking prescription medication for sleeping problems (zolpidem, zopiclone and mirtazapine), eight were on analgesics (primarily paracetamol), five were prescribed sedatives (primarily oxazepam) and three were on antidepressants (escitalopram, citalopram and venlafaxine). Naltrexone was prescribed for one participant with alcohol-related problems. Four of the 17 participants had no ongoing psychopharmacological medication. According to M.I.N.I. two participants had an ongoing depressive episode prior to treatment, one participant had ongoing panic disorder, and one participant had alcohol use disorder. Twelve of the participants had suffered earlier episodes of depression. According to the M.I.N.I, suicide risk was considered moderate for one participant, low for four participants and the remaining 12 were scored as having no risk. Measured with Hospital Anxiety and Depression Scale [40] prior to treatment, participants in general reported low levels of anxiety (M = 6.8, SD = 3.82) and depression (M = 4.5, SD = 2.72). No participants showed major cognitive decline prior to treatment (MMSE: M = 28.7, SD = 1.12).

### The existential group treatment

The existential group treatment was developed in 2017 by the two first authors as a psychological treatment for older adults with psychological distress in primary care. Based on the central assumption of existential therapy that difficult issues will not be resolved by being avoided, the treatment focuses on alleviating experiential avoidance and helping older adults to address and handle existential issues related to aging. Experiential avoidance is defined as an unwillingness to remain in contact with distressing emotions, thoughts, memories, and physical sensations, even when such avoidance creates harm in the long run [41]. The existential group treatment focuses on helping participants gradually approach existential concerns in a systematic way, aiming to find new and more productive ways of handling the existential process of aging.

The basic concept of the existential group treatment is highly inspired by the work of Irvin Yalom [42] who identified four givens of human existence: death, freedom, existential isolation and meaninglessness. Yalom presents a systematic approach to how patients’ relationships to these four concerns may contribute to psychological distress in terms of specific avoidance patterns. While avoidance may alleviate anxiety in the short term, it may hinder personal development and well-being in the long run. Although Yalom also have provided a framework for group therapy [43], the existential group treatment conducted in this study has not been developed in particular to his group therapy work but mainly in relation to his work on existential therapy.

The existential group treatment in this study was offered in weekly group sessions held over 7 weeks, consisting of 4–6 participants and 2 therapists in each group. Every group session lasted 90–120 min including a short coffee break. The existential group treatment followed a structured format over all 7 sessions, presented in Table 1.

**Table 1:** Treatment overview

Session	Session purpose
1. Story of life	Starting the treatment. Participants are presented to each other in relation to personal age-related challenges that they want to focus on during treatment. Participants' ways of handling current and past difficulties in life and existential challenges are explored through a lifeline exercise.
2. Living with or against life	Focus of treatment is deepened, encouraging active exploration of existential challenges as part of life rather than something that needs to be avoided.
3. Freedom	Approaching the existential concern of freedom, reflecting on agency and ways of handling limitations in life.
4. Loneliness	Approaching the existential concern of loneliness, reflecting on isolation and ways of handling relations.
5. Death	Approaching the existential concern of death, reflecting on the finitude of life and ways of handling the limits of time.
6. Towards the end	Approaching the end of treatment, summarizing helpful ways of handling difficulties in life explored during the treatment and formulating what oneself wants to do within the remaining part of life.
7. Ending	Concluding the treatment, reflecting on ways to maintain experiences from the treatment and formulating strategies for doing what is important in life according to oneself.

The treatment is accompanied by working material, to be used both in- and between sessions, consisting of literary texts and therapeutic assignments emphasizing the existential dimensions of aging. First, reflections from earlier sessions are brought up, and a new theme is introduced with a written introduction and inspiration from classical literature. After reading the text, group members are encouraged to express their own experiences and ways of dealing with the theme of the day. By initially focusing on group participants’ own salutogenic factors of coping with previous life stressors through the theme Story of Life, the existential group treatment proceeds by systematically focusing on problems and possibilities related to aging from the existential themes of freedom, loneliness, and death. Although the existential themes are universal aspects of being human, they quite often provoke strong emotions and avoidance patterns– which sets the task of the two therapists to support participants in handling the existential dimensions of life in different ways. Rather than finding a ‘right’ way, an important goal of the treatment is to raise awareness of various potential ways of dealing with the existential dimensions of aging.

### Procedure

All 18 patients who had completed the existential group treatment during 2023 in the first round of the randomized controlled trial, were later contacted after 3–5 months and asked if they wished to participate in an interview about their experiences of the treatment. They received verbal and written information about this qualitative study via the research staff. All but one of the 18 eligible participants agreed to participate. The sample size was determined based on the number of eligible participants, with the aim of providing both depth and diversity in relation to the research question, as well as aligning with common recommendations for sample size in thematic analysis [44]. The interviewer was a final-semester psychology student who underwent training by experienced psychologists in conducting interviews with older adults with mental health problems and was supervised by the research staff throughout the period of data collection. The interviewer had no prior relationship with the participants. To build trust, each interview was given sufficient time to be conducted thoughtfully and respectfully, with a clear focus on each participant’s personal experiences. The interviews took place at the participant’s home or at the primary care center, depending on the choice of the participant. Interview duration was approximately 1.5 h including a coffee break. Participants gave written consent to participate and were informed that their participation was voluntary and that they were free to end their participation at any time without any further explanation. They received no compensation. Interviews were recorded and transcribed by a professional transcription service. The study was approved by the Swedish Ethical Review Authority (2022–02971-01 and 2023–03104-02).

### Semi-structured interviews

The interview guide, shown in the supplementary material, was semi-structured and included questions on participants’ experiences of the existential group treatment, experiences of change and views on what could be seen as pathways to change in the treatment. Questions were open-ended and follow-up questions were used to gain a deeper understanding of the participants’ experiences and to ensure that the interviewer had correctly understood the participants’ responses. To make the participants feel comfortable and relaxed, the interviews were conducted in an informal atmosphere. In one part of the interview the participants were asked to elaborate more directly on whether own engagement and expectations, the group situation, the therapists, the working material, and the existential themes affected their therapeutic process, reflecting the deductive concepts of Wampold’s pathways to change. The interviewer used the working material from the existential group treatment and flashcards as visual aids accompanying some of the questions.

### Analyses

Thematic analysis was used in this study according to Braun and Clarke [45], enabling a flexible approach to data with both deductive and inductive procedures in the coding process. The analysis was done in a two-step coding process with independent parallel coding by the two first authors. In the first step, all transcribed interviews were read by each of the two first authors to get familiarized with the material and data were then coded in relation to the deductive concepts of Wampold’s three pathways to change [34]. In the next step, data representing each deductive coding concept were inductively coded to represent the most meaningful segments within the concepts. This process was done independently by the two first authors to minimize the risk of relevant data getting lost. The inductive codes were then compared to form an initial theme structure by consensus. After this coding procedure, all the authors met to discuss the initial themes, the theme structure, and then revised the themes together in relation to the initial codes. By this procedure all themes that emerged in the analysis were connected to Wampold’s Contextual Model, expressing the participants’ experiences of pathways to change in the existential group treatment.

### Reflexivity

Thematic analysis is a method, where underlying theories and researchers’ subjective positions are understood to affect the analysis [45]. With the two first authors being clinical psychologists in primary care as well as responsible for the development of the existential group treatment, both clinical preconceptions and theoretical understandings of existential therapy can potentially influence the analysis. Prior to data collection, the two first authors were therapists in one of the groups and supervised the therapists who conducted the other groups. An important aspect of the working process was therefore to take advantage of the range of competences among all authors, in gerontology, psychiatry, clinical health care and academia, enabling an understanding of the data both in relation to existential therapy as well as from a wider understanding of older adult patients in the context of health care. To conduct a coherent thematic analysis, a reflexive stand and discussions about preconceptions were maintained throughout the analysis and theories were attempted to be made explicit rather than affecting the analysis in an implicit way. With a theory driven approach, the analysis aims to understand the participants’ experiences in a relevant framework for psychotherapy research, making Wampold’s Contextual Model explicit in the analysis. Although deductive procedures in thematic analysis have the potential to produce rich, complex and useful analysis [45], thoughtful considerations were made throughout this study to avoid possible pitfalls of narrowing a fuller analysis because of the use of pre-existing theory.

## Results

The results of the thematic analysis on how pathways to change are expressed in older adults’ experiences of existential group treatment are presented in Table 2. The table shows the three deductive coding concepts of (1) The pathway to change through expectations (2) The pathway to change through the real relationship (3) The pathway to change through the specific ingredients, and the related themes to each concept based on the interviewed participants experiences of the existential group treatment. Each coding concept is then briefly presented and followed by the themes with illustrating quotes from the participants’ experiences.

**Table 2:** Deductive coding concepts and the themes related to each concept

Deductive coding concepts	Themes
1. The pathway to change through expectations:	1.1. Commitment matters
2. The pathway to change through the real relationship:	2.1. Engaging with others
	2.2. Developing new and different relationships
3. The pathway to change through the specific ingredients:	3.1. Dare to talk about life and death
	3.2. Letting myself be old
	3.3. Making the most of life despite difficulties

### The pathway to change through expectations

The pathway to change through expectations are those factors in a therapeutic process that influence how participants experience the treatment as something that instills hope and that change is possible if committing to the tasks of the treatment [34]. We found one theme for the coding concept of the pathway to change through expectations: Commitment matters.

#### Commitment matters

This theme captures participants’ experiences of making the choice to commit to treatment and how commitment affected their therapeutic process. Among the participants there were various expectations before the start of the existential group treatment and this theme describes how their expectations were transferred into commitment as a pathway to change during the treatment.

When asked about expectations, many participants described an interest in meeting other older adults and found it relevant to engage in existential themes as the topic of the treatment. As one participant put it:

“I thought it seemed a bit interesting, and I was a bit curious about what would come of it. Partly about what kind of questions would be asked. But it was organized so that there was a theme for each time we met. It was also interesting to meet the other women there, who were about the same age. I was probably the oldest one there.”

A factor that seemed to affect participants’ commitment to treatment was related to somewhat ambivalent attitudes among many participants in getting treatment for psychological distress. Some participants described a clear connection between being in distress and participating in the existential group treatment as a way to relieve their suffering. However, several of the participants did not initially view themselves as patients taking part in a treatment, where some rather perceived their participation as taking a course or as making a contribution to science. This was manifested in the form of a general ambivalent attitude towards seeking help for mental health problems, where several participants described positive expectations in taking part in the treatment but vague expectations in general in relation to change due to treatment. One participant said:

“I had expectations, like, that it would be about our shared situation, problems, and things like that… I thought it would be helpful. That it would be positive, both spending time there and also getting a sense of how others handle things and feeling a sense of recognition. However, how much it would change me going forward, I don’t know. I can’t really think about how I thought back then, I might have thought more about the here and now.”

With a general positive attitude toward taking part in the existential group treatment but with somewhat ambiguous expectations in relation to the concept of ‘getting a treatment’, many participants emphasized the importance of committing to the process in order to make it beneficial for themselves. One participant described the importance of commitment in the following way:

“I believe that for many a group like this can be very important, if you’re prepared to open up and willing to share yourself. Because I think you have to do that.”

Some participants expressed a process where their commitment developed over time, from an initially passive position to a more committing attitude. Although skeptical and reluctant at first, one participant described a change in attitude, in order to make the treatment beneficial for her:

“Of course, it matters if you care, and that I left behind this haughty attitude and agreed to being one in the group and that I would participate. Anything else would have been dishonest.

Central to the process of commitment were, for many participants, the relational aspect of the group format, where many described an increased own commitment when seeing other group members commit to the existential group treatment:

“I think that it means a lot that you actually make something out of the situation… like when another participant brought up something that you might not tell everyone, then I did the same. So this commitment, that one actually contributes with material for the group, is important and it depends on the participants. There could be a few who are quiet and don’t say anything and then not much more will come out of it. But that was not the case.”

### The pathway to change through the real relationship

The pathway to change through the real relationship are those factors in a therapeutic process that affect the patient’s outcome of therapy due to the fact that a real relationship emerges in therapy [34]. We found two themes for the coding concept of the pathway to change through the real relationship: Engaging with others and Developing new and different relationships.

#### Engaging with others

Many participants expressed an experience of finding it helpful to listen to others. By being open to other’s perspectives, participants described a process of engaging with others both in an intellectual way of getting new perspectives, as well as in a more relational and experiential way of experiencing others in a similar situation.

Many described a relational experience of realizing that others struggle with similar issues as oneself and one participant struggling with suicidal thoughts described a process of getting more nuanced perspectives on suicide after listening to other participant’s perspectives on the theme:

“You’ve got a little different, what’s it called, perspective on the whole thing. A bit of other input that has made you think it over once more.”

When being open to other group members, many participants seem to have had an experience of feeling less alone. This entailed a process of realizing that one’s struggles are relatable and recognizable in the experiences of others, rather than being seen as a private and personal flaw. As described by the following participant:

“You had a lot in common that you could talk about, and it gave everyone something that you could share how you had lived. And it was very similar. Then you become aware that, well, you’re not alone. There were many more than just me who had it that way. This calmed you down a little.”

When engaging with other group members, many participants described helpful group processes where one participant’s sharing of honest and personal experiences of aging were met with an increased willingness from others to engage in similar meaningful disclosures. Self-disclosures also seemed to promote feelings of kindness and compassion from other group members. Such a process was described by the following participant who contrasted her prior experience of being interviewed by a psychologist with her experience of taking part in the existential group treatment in the following way:

“Yes, the difference was that then it was just me, and he didn’t tell anything in return; it was just questions, so to speak. But in this group, everyone spoke. And the strange thing is that you might not speak at first, and then you wait a little, and then more and more comes. And this woman, who was really depressed and openly said ‘I don’t want to live anymore’, that she could talk and share her tough moments, I thought that was amazing. And it also made the other three of us open up too.”

To many participants, engaging with others seemed to have been an empowering process, where being able to relate to others also made them feel more capable of handling their own difficulties in life. As one participant put it:

“I’ve been thinking about what we’ve talked about and that you’re not alone, above all. There are others who has it like this and are managing it well, then I should be able to do it too.”

While many participants described the group process as being helpful and felt surprised about how close they came to other group members in a short period of time, all did not share this experience. In this context, several participants stressed the therapists’ role in facilitating the group’s exploration of existential themes and maintaining focus on these topics. Hinders to engaging with others seemed to center around negative experiences of group dynamics such as too few participants in a group, apparent cognitive decline among one group member, and when therapists were too vague in leading the group to a joint exploration of existential themes. One participant described disengagement in the process due to lack of therapeutic directiveness and an experience of low engagement from other group members that affected him negatively in the following way:

“Since you did not know where you were heading, we just sat there like isolated islands in this formation. Each one of us had our own story but there was no one who was interested in changing it all.”

When the group dynamic was satisfactory and when group members shared their real-life situations related to aging, a feeling of togetherness seemed to emerge by the process of engaging with others in the existential group treatment. In line with this, one participant described a process of feeling more calm and less worried due to the existential group treatment:

“Maybe it’s because of that, since I feel that I have a greater inner peace than I had before. And that it was, during the time the group was ongoing, very comforting to talk about this and get input from others, that’s probably the most important thing in a way. So alike we are, though different.”

#### Developing new and different relationships

The second theme of the pathway to change through the real relationship that was expressed in the participants’ experiences of relating to others was the development of friendships. When engaging with other group members, many participants described a process of developing new and different relationships with group members which they maintained after treatment. In this study’s data, consisting of participants from five different treatment groups, members from four of the five groups described ongoing contacts with former group members ranging from telephone calls to regular group meetings three months after treatment. One participant described this process as following:

“We’ve met after the group, and we’ve had lunch three times, and it’s been super nice. So, it is just as if one has got three new friends and it feels like you know each other, even though we don’t. It’s been open and good, and we can talk about what we’ve talked about here. I don’t think anyone feels like they can’t talk.”

Many participants described an openness between group members regarding important issues related to aging that seems to have the possibility to nurture friendship even though the group format is relatively short. When engaging with important issues that one doesn’t necessarily speak with others about, a special and different bond between group members seems to have the ability to emerge in existential group treatment that may foster the development of friendship in a certain way. In this sense the development of friendship has to some participants, been shaped by their mutual experience of engaging with important issues related to aging with an openness they have tried to contain in their development of friendship after the treatment has ended. One participant described this process in the following way:

“Suddenly, we know a lot about each other. It makes interaction easy and effortless. And as I mentioned at the beginning, these are not things you bring up unless someone leads you directly in the group. This is a very strong reason to continue with this type of program.”

### The pathway to change through the specific ingredients in existential group treatment

The pathway to change through the specific ingredients, are those factors, apart from expectations and relational factors in a specific type of therapy that promote psychological health [34]. In relation to the participants’ experience of existential group treatment we found three themes related to this concept: Dare to talk about life and death, Letting myself be old and Making the most of life despite difficulties.

#### Dare to talk about life and death

Many participants described a process of being able to talk about difficult issues as a part of the existential group treatment. To be able to speak of existential dimensions of life such as death, loneliness and sufferings, were described as being important and helpful by many participants and were also something that some emphasized were not easy to talk about in their everyday life due to fears and possible tabus in relation to these topics. One participant described an experience from the existential group treatment of feeling more at peace, which she attributed to the ability to talk about death:

“There has come a sense of calmness after this. And I also think, this thing about having talked so openly about death there, because I can’t talk about the fact that I’m going to die with my children.”

Many participants described a genuine willingness to talk about difficult issues of life in existential group treatment, whereas some on the other hand described an initial opposition of approaching these topics. When the format of the existential group treatment then encouraged an exploration of topics that one doesn’t naturally talk about, it was experienced as emotionally demanding by some. When further engaging in the process of daring to talk, the ones who decided to let go and approach their sensitive topics of life and aging despite initial reluctance described an experience of relief as well as being able to leave things behind afterwards. One of these participants described this process of talking about difficult issues of life in the existential group treatment in a way she hadn’t done before, in the following way:

“You’ve opened up. Because I had really locked that door, and when we talked about it and had that discussion, it somehow felt very comforting. Because there were some things I didn’t share with my friends or anything like that.”

Since difficult issues of life and existential themes per definition can be difficult to talk about, many of the participants described the importance of the therapists and the working material to stay on the path regarding these existential topics. In relation to this, many participants found the existential material relevant to their own situation, however some found it a bit too pompous or complicated and described a wish to balance it with more positive material on aging.

When participants from own personal willingness, and with different levels of initial reluctance dared to talk about difficult issues of life and death, a pathway to change was apparent in the material. One participant with a close relative who was dying, summarized how the existential group treatment influenced her ability to talk about difficult life issues in the following way:

“I really worked with it. And I know that when we came to death, I thought no, this I will skip. That’s what I thought at first. No, I can’t handle that, being alone and going through that. Well, then I started reading a little, and then a little more… So, I cautiously went on, and then [exhales]. Well, now I even can, when my close relative throws something out like that, last time she said, ‘Why should one have an expensive coffin when there are blankets?’ And then I said ‘No, that is completely right’. I never would have been able to answer her if I had not gone through this. I never would have, it would have gotten stuck there.”

#### Letting myself be old

Some participants described a pathway to change that involved the development of a more accepting attitude towards aging. This process, of accepting one’s own life situation for what it is, was for many related to talking about one’s own and other’s current life situations, their stories of life, as well as the existential themes at the core of the existential group treatment. One participant emphasized this insight as ‘letting myself be old’:

“Overall, opening up and admitting that one is old. And admitting to myself that I am old. I have had a really hard time with that… Because in order to admit, I first have to start with myself. I can’t expect something to happen if I don’t accept it myself. If everyone who is old don’t accept that they are getting older and no longer function properly, how can one then be helped?”

In some sense the ability to talk about difficult life issues and to focus on the challenging parts of one’s own life situation, seemed for several participants to have led to a more accepting attitude towards their life situation in general. For some this was related primarily to accepting their current life stage and for some it was manifested in a more accepting attitude towards the existential dimension of life in general. One participant described her new thoughts in the following way:

“The new thoughts were really about accepting that it’s a change… I have a greater acceptance of aging. That it kind of means a new phase … A shorter, well, a more clear focus on the fact that it’s a limited time.”

Another participant described a more emotionally demanding process of developing an accepting attitude towards aging. As part of the group treatment, she began to think about a difficult period in her life that she hadn’t thought about for a long time. She described how this troubled her, using her own terminology, as ‘flashbacks’ during a period of the existential group treatment. Even though it was described as a demanding process for the participant to think about past difficulties, she also described it as the most important part of the treatment for her, and that bringing up these difficulties in her life also made her more serene with her current life situation and the struggles she been through:

“It has taught me so much. It taught me about the flashback that made me sad and down. But it also taught me to accept the life I have now and to think that it’s good.”

The pathway to change through the specific ingredients of existential group treatment, in developing an accepting attitude towards aging, seemed to entail a process among participants to encounter some demanding aspects of life that hasn’t been incorporated as apparent in their life situation before and to accept this as a part of life. An example of this process could be seen where a participant contrasted her previous understanding of death with her new thoughts after the existential group treatment in the following way:

“Mostly like a complete, sort of like outer space. I can’t grasp it, and of course, I can’t. So, it was completely incomprehensible. Now, it’s more of an acceptance. Yes, it’s going to happen. And now it’s more, in what way is it going to happen?”

#### Making the most of life despite difficulties

A third pathway to change through the specific ingredients was described by some participants as an increasing will to make the most of life despite age-related difficulties such as loss of loved ones, declining physical health and loneliness. This was manifested in various ways, such as an increased will to engage in social contacts and family, prioritizing meaningful activities, deciding to travel, and taking time for practical necessities that had previously been postponed. As one participant described:

“Well, if we think about what has given me something, it’s that I’m able to seize opportunities, focus more on what I can make of my life. So, a more active attitude, that I don’t just have to sit and think that I’m old and forgetful. During the time my husband was sick, we didn’t travel, and I thought like this, now it’s time. So, I’ve been to Rhodes.”

For some participants, the process of making the most of life seemed related to their exploration of the existential themes of death and freedom, and the emphasis on the fact that everyone struggles with the task of taking own responsibility in life, even though there are difficulties and time is limited. With the ability to talk about death and own responsibility in relation to the existential theme of freedom, some participants described an increased determination of making the most of life due to the existential group treatment:

“I think it has made me feel that I really want to seize the moment, and I really want to stay active. And I feel that after I turned eighty it’s just like, now is the time to make the most of it, because you don’t know how much time you have.”

Another aspect of Making the most of life despite difficulties, were how some participants related this process to the process of letting oneself be old. Even though there were some participants who primarily described an increased eager in doing more meaningful things as a result of existential group treatment, there were also participants who manifested this process in relation to an accepting attitude towards aging where they wanted to adapt to their current life situation in a meaningful way:

“I think it has to do with accepting the situation you’re in now. And I can’t get my husband back, so I can’t just go around thinking ‘Oh God, poor me’. Instead, I have to make the best of it now.”

In relation to Dare to talk about life and death, as well as Letting myself be old, the third pathway to change in Making the most of life despite difficulties, further manifested a will among some participants to take responsibility and increased agency in life. As one participant put it:

“The firm conclusion I came to is that I’ve decided to speak up about what I need. That’s a very big thing. It wouldn’t have happened if I hadn’t participated in this experiment. And it goes a long way, too.”

## Discussion

Findings suggest that all three of Wampold’s pathways to change were of relevance for the therapeutic process within existential group treatment for older adults with psychological distress. These pathways may help explain how existential therapy works and potentially benefits this age group. In previous research, a few qualitative studies have evaluated participants’ experiences of existential interventions [25–27] but to our knowledge, no qualitative study has specifically explored older adults’ experiences of existential therapy within primary care or analyzed experiences of existential therapy in relation to an established psychotherapeutic model of change.

In terms of expectations that the group treatment would bring about change, participants expressed a somewhat ambivalent attitude. In a recent systematic review, older adults have been shown to be less likely to seek professional help and may prefer informal mental help over professional help due to possible stigma and negative beliefs about mental health services [46]. Similar barriers may possibly have influenced participant’s expectations in this study, where expectations were generally positive but centered mainly around taking part in a group setting rather than undertaking a specific treatment for psychological distress. The expectations among the participants were in this sense mostly relational based with a primary focus on getting new perspectives from other participants in similar life situations concerning the existential dimensions of aging. Treatment for a specific disorder has traditionally not been a pivotal focus of existential therapy [47], and this may further have accentuated patients’ explorative stance rather than creating expectations that the treatment would diminish their psychological distress.

Salient is also the importance of relational factors that older adults expressed as a pathway to change in existential therapy. The two themes Engaging with others and Developing new and different relationships reflect the relational pathways to change in existential group treatment. Although several participants emphasized the importance of the therapists in leading the group to a joint exploration of existential themes, the relational factors affecting change seemed mostly to center around the experiences of engaging with others. In group treatment, group cohesion, in terms of participants’ relationship with other group members and with the group as a whole has been related to outcome [48]. Central aspects of relational factors in individual therapy according to Wampold’s Contextual Model, such as empathy, warmth and collaboration [34], was in this sense mainly manifested within a group context in this study. Given the well-established association between loneliness and mental health problems [12, 49], and the fact that the vast majority of participants in this study lived alone, it is important to highlight that many participants described a process of making friends by staying in contact with one another after existential therapy. It seems like existential group treatment may have a way of stimulating a deepened engagement with others characterized by a mutual and trustworthy exploration of existential themes which many found to differ from their everyday relationships and possible to continue after treatment. Central to this process, as many participants put it, was the importance of other group members’ brave sharing of sensitive disclosures. This, in turn, helped participants engage further in the treatment and grow closer to others through existential group treatment. In relation to Wampold’s contextual model, where the therapeutic relationship is seen as the foundation upon which specific therapeutic ingredients operate [34], the experiences of older adults in this study suggest a mutual enrichment between relational and existential factors during the process of existential group treatment.

In understanding older adults’ experiences of the specific ingredients in existential group treatment: Dare to talk about life and death, Letting myself be old, and Making the most of life despite difficulties, were generated as central themes defining existential group treatment. With the aim of the existential group treatment developed by Erling and Waltersson to help older adults to address and handle existential issues related to aging by gradually alleviating experiential avoidance, it is important to stress how the themes Dare to talk about life and death and Letting myself be old, seems to encapsulate this process. In some sense, the ability to talk about life and death and finding the courage to approach existential dimensions of one’s own life despite possible reluctance, seems to be an important first step of engaging in the basic existential premise that difficult issues will not be resolved by being avoided. By encountering the existential dimensions of one’s life in a safe and sincere group setting with others, a more accepting attitude toward one’s life situation, as a way of letting oneself be old, can emerge. These processes may possibly be understood as ‘experiential acceptance’, in line with Vos’ [28] understanding of the key mechanism of change in existential therapy being to help clients live a meaningful life where one can cope with limitations and challenges by not ignoring nor denying the totality of one’s experiences. With the concept of experiential avoidance rooted in the psychological flexibility model, previous research has highlighted possible similarities between existential therapy and Acceptance and Commitment Therapy, where possible change in psychological health or well-being during existential therapy may be mediated by psychological flexibility [50]. This underscores the importance of future research examining the role of experiential avoidance, within the framework of the psychological flexibility model, as a potential mediator in quantitatively measured outcomes of existential therapy.

When seeing things for what they are and being able to confront the reality of aging, a desire to make the most of life despite difficulties can emerge from existential therapy. Participants in this study described changes made in their everyday life after treatment, such as an increased will to engage in social contacts and prioritizing meaningful activities, which may lead to possible health benefits over time. A coming publication will expand this line of research and try to answer the quantitative question if these pathways to change, that’s been expressed in the experiences of older adults after completing existential group treatment, can lead to measurable changes in psychological distress and quality of life [35].

### Limitations

All participants in this study were recruited from an ongoing randomized controlled trial [35], with inherent processes related to recruitment and screening which may not fully reflect a naturalistic primary care setting. Due to randomization, group members were not clinically selected to optimize group dynamics and groups were relatively small. A possible limitation of deductive procedures in thematic analysis is the potential narrowing of a fuller analysis due to the use of pre-existing theory [45]. To mitigate this, meaningful segments of the data not directly related to Wampold’s Contextual Model were systematically considered in the analysis, and parallel coding was used to ensure that codes and themes were well grounded in the data. An important aspect of the data collection was also the use of an external interviewer. To ensure that all eligible participants who completed the existential group treatment had the opportunity to share their experiences, all participants were invited for interviews. With regards to data saturation, additional insights could possibly have emerged from a larger sample but based on common sample size recommendations and ongoing discussions between the authors during the analysis, it was considered unlikely that more interviews would bring new themes or further enhance complexity and variety to the analysis. A final consideration is that the participants in this study had a relatively high level of education, and findings cannot be directly extrapolated to older adults with lower educational backgrounds.

### Clinical implications

Older adults’ experiences of existential group treatment suggest that engaging in existential dimensions of aging can be helpful when done in a group format together with others. Various mental health problems within the concept of psychological distress seem to be tolerated by group members, but special group considerations such as avoiding too small groups and apparent cognitive decline is important in order to enable good group dynamics. Since some participants found it troublesome when group members exhibited signs of cognitive impairment, it may be suggested that existential group treatment is not suitable for older adults with apparent cognitive decline. Overall, the participants’ descriptions of pathways to change suggest that existential group treatment could provide a meaningful addition in the primary care services for older adults with psychological distress. A further clinical implication could also be the possibility of other psychotherapeutic approaches for older adults to incorporate existential interventions, in line with how pathways to change have been expressed by participants in this study. Given the issues of loneliness and its relation to psychopathology, the fact that many participants developed new social connections through treatment when engaging in the existential dimensions of aging together with others suggests that existential group treatment may have clinical relevance for addressing these problems as well.

## Conclusion

The present study has made the process of existential therapy understandable through Wampold’s three pathways to change and has emphasized the possible role of experiential avoidance in existential group treatment for older adults with psychological distress. Our results suggest that engaging in the existential dimensions of aging can be helpful when done in a structured setting together with others and has documented pathways to change in existential group treatment that may lead to possible health benefits for older adults with psychological distress.

## Supplementary Information

Supplementary Material 1.

## Data Availability

The data presented in this article are not readily available because the transcripts cannot be sufficiently de-identified to ensure participant anonymity, making it unsuitable for sharing. Requests to access the data should be directed to isak.erling@gu.se.
